# Rare, evolutionarily unlikely missense substitutions in *CHEK2 *contribute to breast cancer susceptibility: results from a breast cancer family registry case-control mutation-screening study

**DOI:** 10.1186/bcr2810

**Published:** 2011-01-18

**Authors:** Florence Le Calvez-Kelm, Fabienne Lesueur, Francesca Damiola, Maxime Vallée, Catherine Voegele, Davit Babikyan, Geoffroy Durand, Nathalie Forey, Sandrine McKay-Chopin, Nivonirina Robinot, Tù Nguyen-Dumont, Alun Thomas, Graham B Byrnes, John L Hopper, Melissa C Southey, Irene L Andrulis, Esther M John, Sean V Tavtigian

**Affiliations:** 1 International Agency for Research on Cancer, 150 Cours Albert Thomas, Lyon CEDEX 08, F-69372, France; 2 Laboratory of Cancer Genetics, Center of Medical Genetics and Primary Health Care, 4 Tigran Mets Avenue, Yerevan 375010, Armenia; 3 Department of Internal Medicine, University of Utah School of Medicine, 391 Chipeta Way, Suite D, Salt Lake City, UT 84108, USA; 4 Centre for Molecular, Environmental, Genetic and Analytic Epidemiology, The University of Melbourne, 723 Swanston Street, Melbourne, Victoria 3010, Australia; 5 Cancer Care Ontario, Samuel Lunenfeld Research Institute, Mount Sinai Hospital, Department of Molecular Genetics, University of Toronto, 60 Murray Street, Toronto, ON M5T 3L9, Canada; 6 Cancer Prevention Institute of California, 2201 Walnut Avenue, Suite 300, Fremont, CA 94538, USA; 7 Department of Pathology, The University of Melbourne, Medical Building 181, Melbourne, Victoria 3010, Australia; 8 Stanford University School of Medicine, 300 Pasteur Drive, Stanford, CA 94305, USA; 9 Department of Oncological Sciences, Huntsman Cancer Institute, University of Utah School of Medicine, 2000 Circle of Hope, Salt Lake City, UT 84112, USA

## Abstract

**Introduction:**

Both protein-truncating variants and some missense substitutions in *CHEK2 *confer increased risk of breast cancer. However, no large-scale study has used full open reading frame mutation screening to assess the contribution of rare missense substitutions in *CHEK2 *to breast cancer risk. This absence has been due in part to a lack of validated statistical methods for summarizing risk attributable to large numbers of individually rare missense substitutions.

**Methods:**

Previously, we adapted an *in silico *assessment of missense substitutions used for analysis of unclassified missense substitutions in *BRCA1 *and *BRCA2 *to the problem of assessing candidate genes using rare missense substitution data observed in case-control mutation-screening studies. The method involves stratifying rare missense substitutions observed in cases and/or controls into a series of grades ordered *a priori *from least to most likely to be evolutionarily deleterious, followed by a logistic regression test for trends to compare the frequency distributions of the graded missense substitutions in cases versus controls. Here we used this approach to analyze *CHEK2 *mutation-screening data from a population-based series of 1,303 female breast cancer patients and 1,109 unaffected female controls.

**Results:**

We found evidence of risk associated with rare, evolutionarily unlikely *CHEK2 *missense substitutions. Additional findings were that (1) the risk estimate for the most severe grade of *CHEK2 *missense substitutions (denoted C65) is approximately equivalent to that of *CHEK2 *protein-truncating variants; (2) the population attributable fraction and the familial relative risk explained by the pool of rare missense substitutions were similar to those explained by the pool of protein-truncating variants; and (3) *post hoc *power calculations implied that scaling up case-control mutation screening to examine entire biochemical pathways would require roughly 2,000 cases and controls to achieve acceptable statistical power.

**Conclusions:**

This study shows that *CHEK2 *harbors many rare sequence variants that confer increased risk of breast cancer and that a substantial proportion of these are missense substitutions. The study validates our analytic approach to rare missense substitutions and provides a method to combine data from protein-truncating variants and rare missense substitutions into a one degree of freedom per gene test.

## Introduction

Familial clustering of breast cancer is well recognized, having been described over 140 years ago [1]; the familial relative risk of breast cancer is on average about twofold and is higher among relatives of patients with early-onset cases [2,3]. Three classes of breast cancer susceptibility sequence variants with different levels of risk and prevalence in the population are now well established [4,5]: rare high-risk variants, such as protein-truncating mutations in *BRCA1*, *BRCA2*, *PTEN *and *TP53 *(Mendelian Inheritance in Man numbers (MIMs) 113705, 600185, 601728 and 191170, respectively); rare intermediate-risk variants, such as protein-truncating mutations in *ATM *[6,7], *BRIP1 *[8], *CHEK2 *[9] and *PALB2 *[10,11] (MIMs 208900, 605882, 604373 and 610355 respectively); and, more recently, common modest penetrance variants such as the risk single-nucleotide polymorphisms (SNPs) detected by genome-wide association studies (GWASs) in *FGFR2*, *TOX3 *(*TNRC9*), *MAP3K1 *and *LSP1 *[12-14] (MIMs 176943, 611416, 600982 and 153432, respectively). High-risk variants in the known major breast cancer susceptibility genes *BRCA1*, *BRCA2*, *TP53 *and *PTEN *account for approximately 20% to 25% of the familial risk of breast cancer, and adding the known intermediate-risk genes increases the proportion by perhaps 1% for each gene [15]. Moreover, the panoply of known modest-risk SNPs account for about 8% of the familial relative risk [16]. Thus known genetic effects account for about one-third of the familial relative risk of breast cancer, leaving two-thirds unaccounted for, a phenomenon referred to as the "problem of missing heritability." Some of this so-called missing "heritability" is of course due to the familial component of environmental risk factors; the measured surrogates for these factors probably explain about 5% of the familial relative risk, but if measured more specifically and more precisely, they may explain considerably more familial aggregation [17].

The gene *CHEK2 *encodes a serine/threonine kinase, CHK2, that functions in the signaling pathways activated by DNA damage, particularly DNA double-stranded breaks [18]. Inheritance of a *CHEK2 *protein-truncating mutation such as the relatively well investigated Northern European founder mutation *c.1100delC *confers a two- to threefold increased risk of breast cancer, an increased risk of a number of other cancer types and perhaps a decreased risk of some smoking-related cancers [9,19-21]. Some missense substitutions in *CHEK2 *also alter cancer risk, as exemplified by the Ashkenazi *CHEK2 *missense substitution p.S428F and the Slavic substitution p.I157T [22-26]. Most large-scale genetic studies of *CHEK2 *conducted to date have focused on genotyping known variants, such as founder mutations. Consequently, there has been little opportunity to assess the role of the potentially more numerous, rarer variants of this gene.

During the 1990s, linkage analysis proved to be an effective genome-wide approach for finding high-risk susceptibility genes for breast and colon cancer. Over the past few years, GWASs have proved to be an effective genome-wide approach to finding common, not necessarily causal, SNPs associated with modest risk. Case-control mutation screening, or its quantitative trait homolog of comparative mutation screening of individuals from the opposite ends of a trait spectrum, is emerging as a useful strategy for identifying and characterizing intermediate-risk susceptibility genes [6-8,10,27-29]. While case-control mutation screening has been, to date, too technically demanding to examine a whole biochemical pathway, let alone the entire exome, one can imagine combining exon hybridization capture and massively parallel sequencing to accomplish such a study design. Beyond the laboratory challenge imposed by the implied scale of resequencing, a second challenge is to conduct a statistically powerful analysis of the large number of rare sequence variants that would be revealed if such a study design were applied to a common disease such as breast or colon cancer. Previously, we used data from mutation screening of *ATM *in breast cancer patients and controls to demonstrate the ability to detect evidence of pathogenicity from both truncating and splice junction variants (T+SJV) and rare missense substitutions (rMS) [7]. Here we apply the same analytic strategy to *CHEK2 *and then extrapolate the results to determine the requirements for much larger-scale studies.

## Materials and methods

### Ethics statement

The *CHEK2 *mutation-screening studies and analyses described here were approved by the institutional review board (IRB) of the International Agency for Research on Cancer, the University of Utah IRB and the local IRBs of the Breast Cancer Family Registry (Breast CFR) centers from which we received samples. All participants gave written, informed consent.

### Subjects

Patients were selected from among women gathered by population-based sampling by the Breast CFRs at three centers (Cancer Care Ontario, the Cancer Prevention Institute of California (formerly the Northern California Cancer Center) and the University of Melbourne) [30]. Patients were recruited between 1995 and 2005.

Selection criteria for cases (*N *= 1,313) were diagnosis at or before age 45 years and self-reported race or ethnicity plus grandparents' country of origin consistent with Caucasian, East Asian, Hispanic/Latino or African American racial or ethnic heritage.

The controls (*N *= 1,123) were frequency matched to cases within each center on racial or ethnic group, with age at selection not more than ± 10 years difference the age range at diagnosis of the patients gathered from the same center. Because of the shortage of available controls in some ethnic and age groups, the frequency matching was not one-to-one in all subgroups.

### Mutation screening

Mutation screening started from whole-genome amplified (WGA) DNA for coding exons 1-9 and from genomic DNA for exons 10-14. A nested polymerase chain reaction (PCR) strategy was used, followed by high-resolution melting (HRM) curve analysis [31,32] and then dye terminator resequencing of samples that contained a melt curve aberration indicative of the presence of a sequence variant. For *CHEK2 *amplicons harboring SNPs with a frequency ≥1% in either the Single Nucleotide Polymorphism Database (dbSNP) [33] or initial amplicon testing, we applied a simultaneous mutation scanning and genotyping approach using HRM curve analysis to improve the sensitivity and efficiency of the mutation screening [34]. The laboratory process used was the same as that described in detail for our recent case-control mutation screening for *ATM *[7], except that primary PCR assays for *CHEK2 *exons 10-14 (which are involved in a subtelomeric repeat) relied on a long-range PCR assay as described by Sodha *et al. *[35].

All exonic sequence variants, plus splice junction consensus sequence variants that reduced splice junction sequence similarity to the standard consensus sequences AG^GTRRGT (donor) or Y_16_NYAG^ (acceptor) (where ^ indicates the position of the splice junction), were reamplified from genomic DNA for confirmation of the presence of the variant. Because of the presence of pseudogenes that partially matched the sequence of the *CHEK2 *long-range PCR exons (exons 10-14), sequence variants identified within these exons were subsequently tested using allele-specific PCR assays for the primary PCR to confirm that the sequence variants initially identified were true *CHEK2 *variants. To ensure amplification of the *CHEK2 *DNA sequence and not amplification of the potentially interfering *CHEK2 *pseudogenes, the positions of the specific primers were chosen so that the 3' extremity bases perfectly matched the *CHEK2 *wild-type sequence, while they mismatched the corresponding position of the pseudogenes.

All samples that failed at the primary PCR, secondary PCR or sequencing reaction stage were reamplified from WGA DNAs or genomic DNA. Samples that still did not provide satisfactory mutation-screening results for at least 80% of the *CHEK2 *coding sequence were excluded from further analyses (*n *= 24). Process and data management of the mutation screening were carried out as described by Voegele *et al. *[36]. Primer and probe sequences are available from FLCK upon request.

### Alignments and scoring of missense substitutions

Previously, we used the T-Coffee (Tree-based Consistency Objective Function for alignment Evaluation) software suite of alignment tools [37,38] to prepare a CHK2 protein multiple sequence alignment in which the most diverged sequence was from sea urchin (*Strongylocentrotus purpuratus*) to analyze a small number of *CHEK2 *missense substitutions and in-frame deletions [39]. We updated this alignment by replacing the partial pufferfish (*Tetraodon nigroviridis*) sequence with a full-length zebrafish (*Danio rerio*) sequence and including predicted CHK2 sequences from elephant (*Loxodonta africana*), platypus (*Ornithorhynchus anatinus*), tunicate (*Ciona intestinalis*) and fruit fly (*Drosophila melanogaster*). The alignment was characterized by (1) determining percentage sequence identity between each pair of sequences in the alignment, (2) using the Protpars routine of Phylogeny Inference Package version 3.2 software (PHYLIP; free software developed by Felsenstein [40]) to make a maximum parsimony estimate of the number of substitutions that occurred along each clade of the underlying phylogeny and (3) recording the "median sequence conservation score" reported by the missense substitution analysis program Sorting Intolerant from Tolerant (SIFT) [41,42]. The sequence alignment, or updated versions thereof, is available at the Align-GVGD website [43]. Missense substitutions observed during our mutation screening of *CHEK2 *were scored using the Align-GVGD [43-45] and SIFT [41,42] software programs with our curated alignments and with Polymorphism Phenotyping version 2 software, or PolyPhen-2, using its precompiled alignment [46,47].

### Statistical analysis and power calculations

To assess risk associations using the case-control frequency distribution of T+SJVs and rMSs, we constructed a single table with one entry per participant; zero or one rare sequence variant per participant; and annotations for type of sequence variants, study center, case-control status, race or ethnicity, and age. For the two participants who carried more than one rare variant of interest (one participant carried p.I448S (C15) plus p.E394D (C35), and one participant carried p.E239K (C15) plus p.R346H (C25)), only the variant belonging to the more likely evolutionarily deleterious grade (that is, higher C-number as scored by Align-GVGD) was considered.

Most analyses were performed using multivariable unconditional logistic regression using Stata version 11 software (StataCorp, College Station, TX, USA). Differences in the case-control ratio between ethnic groups and age categories were accounted for by including categorical variables for each age category and ethnic group. Adjustment was also made for study center. We explored the possibility of interactions between ethnic group and study center, checking both improvement of model fit by the likelihood ratio statistic and comparing the estimates of the parameter of interest (log odds ratio (OR) per Align-GVGD grade) in different models. Adjustment for ethnic group should also capture confounding of genetic and social factors with interaction terms, allowing that this confounding effect may be different for the broadly labeled ethnic groups in different centers. Because the Breast CFR matched cases and controls for age in 5-year categories, and because the maximum age of Breast CFR patients included in this study was 45 years, all participants ages 41 years and older (at diagnosis for patients and at ascertainment for controls) were combined into a single age category.

Logistic regression trend tests were formatted such that participants who did not carry any T+SJV or any rMS, as well as carriers of the seven grades of rMSs (C0, C15, C25, C35, C45, C55 and C65) defined by Align-GVGD [45], were assigned the default row labels 0, 1, 2, 3, 4, 5, 6 and 7, respectively. These row labels were then used as a continuous variable in the logistic regression analyses. Regression coefficients and trend test *P *values (*P*_trend_) were estimated from the resulting lognormal ORs using the logit function of Stata software. Carriers of T+SJVs were analyzed against the same noncarrier group defined above. Two strategies were used to combine evidence of association with T+SJV and rMS variants: (1) carriers of T+SJVs were combined with carriers of C65 rMSs in category 7, and (2) T+SJV carriers were assigned row label 8. We used the Fisher's exact test to obtain the lower bound of the 95% confidence interval (95% CI) for associations with categories that contained one or more patients but zero controls.

*Post hoc *power calculations were performed by specifying a hypothetical OR and population prevalence for each class of variant, together with the cumulative probability of breast cancer prior to age 70 years. The ORs and control carrier frequencies that we specified for the individual grades of sequence variants, relative to the noncarriers, were based on data from the population-based Breast CFR sample series. For the grades for which there were a reasonable number of observations, that is, C0, C15, C25, C65 and T+SJV, we used the adjusted ORs and observed carrier frequencies. Because of the very low numbers of observations in grades C35-C55, ORs for these categories were estimated from the logistic regression OR coefficient and population carrier frequencies defined to obtain the specified OR, given the number of observations in patients. On the basis of these OR and frequency estimates, we calculated expected values and variances of the test statistics for the types of test considered: Pearson's χ^2 ^test for the two-category tests and the Wald statistic from a logistic regression for the trend test. We then calculated the probability of these statistics exceeding a series of desired *P *value thresholds using a normal approximation.

Attributable fractions were estimated according to the method described by Greenland [48], and familial relative risks were estimated according to the methods described by Goldgar [49]. Both calculations used the same frequency and risk association estimates as those used for the *post hoc *power calculations.

## Results

### Number of subjects included in the analysis

Of the 2,436 Breast CFR participants, 24 (10 patients and 14 controls) were excluded because their PCR failure rate for *CHEK2 *mutation-screening amplicons was greater than 20% (Table 1). The distributions of the remaining cases and controls by age, race or ethnicity, and study center are detailed in Table 2.

**Table 1:** Participants excluded because of poor mutation-screening performance by study center^a^

Center	Patients, *n *(%)	Controls, *n *(%)
Breast CFR Australia	5 (0.8%)	11 (2.1%)
Breast CFR Canada	1 (0.3%)	2 (0.4%)
Breast CFR Northern California	4 (1.0%)	1 (0.7%)
Total	10 (0.8%)	14 (1.2%)

^a ^All 10 excluded patients were <42 years old, and all 14 excluded controls were <45 years old; percentage data are the percentages of the total number of patient or control DNA provided by the indicated Breast CFR center; Breast CFR, Breast Cancer Family Registry.

**Table 2:** Distribution of patients and controls by age, race or ethnicity, and study center^a^

Distributions	Patients, *n *(%)	Controls, *n *(%)
Age range, yr		
≤30	106 (8.1%)	66 (6.0%)
31-35	322 (24.7%)	171 (15.4%)
36-40	434 (33.3%)	231 (20.8%)
41-45	441 (33.8%)	199 (17.9%)
46-50	0 (0.0%)	230 (20.7%)
51-55	0 (0.0%)	212 (19.1%)
Total	1,303 (100.0%)	1,109 (100.0%)
		
Race or ethnicity		
Caucasian	843 (64.7%)	956 (86.2%)
East Asian	204 (15.7%)	70 (6.3%)
Latina	158 (12.1%)	47 (4.2%)
Recent African ancestry	98 (7.5%)	36 (3.2%)
Total	1,303 (100.0%)	1,109 (100.0%)
		
Study center		
Breast CFR Australia	588 (45.1%)	513 (46.3%)
Breast CFR Canada	302 (23.2%)	461 (41.6%)
Breast CFR Northern California	413 (31.7%)	135 (12.2%)
Total	1,303 (100.0%)	1,109 (100.0%)

^a ^Patients and controls excluded because of poor mutation-screening performance are not included; percentage data are the percentages of the total number of patient or control DNA in the category indicated that met the mutation-screening quality control criterion; Breast CFR, Breast Cancer Family Registry.

### Analysis of protein-truncating variants

Full open reading frame mutation screening of *CHEK2 *revealed three distinct nonsense substitutions and four distinct small insertion deletion variants that should result in a truncated protein. One of these, *c.1100delC*, a well-known Northern European founder mutation that has been shown beyond any reasonable doubt to confer a moderately increased risk of breast cancer [50], was observed in 11 patients compared with three controls. The other six protein-truncating variants were observed once each, always in a patient (Supplementary Table S1 in Additional file 1). The overall OR associated with T+SJVs was 6.18 (*P *= 0.005) (Table 3). However, as *1100delC *genotyping has already been reported for most of the Breast CFR participants included in this study [50,51], we note that the combination of the other six protein-truncating variants was marginally significant by itself (*P *= 0.033), but since none of this set of controls were found to carry such a variant, we could not estimate the OR.

**Table 3:** Analyses of rare variants with missense substitutions stratified by Align-GVGD grade^a^

Class	Patients, *n*	Controls, *n*	Crude OR (95% CI)	Adjusted OR (95% CI)
Noncarriers	1,242	1,089		
				
T+SJV	17	3	4.97 (1.45 to 17.0)	6.18 (1.76 to 21.8)
				
Any rMS	44	17	2.27 (1.29 to 4.00)	2.20 (1.20 to 4.01)
				
rMS stratified by Align-GVGD grade^b^				
C0	12	9	1.17 (0.49 to 2.79)	1.39 (0.55 to 3.56)
C15	14	5	2.46 (0.88 to 6.84)	1.82 (0.62 to 5.34)
C25	7	2	3.07 (0.64 to 14.8)	2.47 (0.45 to 13.49)
C35	1	0	-	
C45	0	0	-	
C55	1	0	-	
C65	9	1	7.89 (1.00 to 62.4)	8.75 (1.06 to 72.2)
				
rMS stratified by SIFT grade^c^				
S > 0.05	21	8	2.30 (1.02 to 5.22)	1.99 (0.83 to 4.77)
0.05 ≥ S > 0.00	12	5	2.10 (0.74 to 5.99)	1.91 (0.63 to 5.86)
S = 0.00	11	4	2.41 (0.77 to 7.59)	3.03 (0.91 to 10.0)
				
rMS stratified by PolyPhen-2 grade				
Benign	16	7	2.00 (0.82 to 4.89)	1.69 (0.64 to 4.41)
Possibly D^d^	10	6	1.46 (0.53 to 4.03)	1.65 (0.55 to 4.89)
Probably D^e^	18	4	3.95 (1.33 to 11.7)	3.87 (1.25 to 12.0)

^a ^Odds ratios are adjusted for race or ethnicity (Caucasian, East Asian, African American or Latina), study center, and age as categorical variables; OR, odds ratio; 95% CI, 95% confidence interval; T+SJV, protein-truncating variants plus splice junction variant; rMS, rare missense substitution; S, SIFT score; ^b^Using the *CHEK2 *sequence alignment through *S. purpuratus *(sea urchin); ^c^Using the *CHEK2 *sequence alignment through *D. melanogaster *(fruit fly); ^d^PolyPhen-2 grade "Possibly Damaging"; ^e^PolyPhen-2 grade "Probably Damaging."

### Analysis of rare missense substitutions

In the course of this mutation screening, we observed 34 distinct *CHEK2 *missense substitutions (Supplementary Table S1 in Additional file 1). The majority (24 of 34) of these were observed once each. The most common one, p.I448S, was observed 10 times, and none had an overall frequency greater than 1% in this sample series. Overall, 42 of the patients carried one rMS, 2 of the patients carried two rMSs, and 17 controls carried one rMS. Thus, there was a significant excess of rMS carriers among the patients (OR = 2.20, *P *= 0.010).

To analyze the rMSs in more detail, we prepared and characterized a protein multiple sequence alignment containing CHK2 sequences from seven mammals, three additional vertebrates, two additional deuterostomates and one protostomate. Ordering the nonmammalian sequences by decreasing identity to human CHK2 and sequentially assessing overall sequence diversity, the alignment exceeded a maximum parsimony estimate of an average of three substitutions per position upon inclusion of the sea urchin (*Strongylocentrotus purpuratus*) sequence (Supplementary Table S2 in Additional file 1). Three substitutions per position was suggested as a criterion of sequence diversity for analysis of missense substitutions, and we have adopted it as our criterion for use with Align-GVGD in case-control mutation-screening applications [7,52,53].

Using this alignment, we scored the 34 missense substitutions with Align-GVGD [43-45] and SIFT [41,42] (Supplementary Table S1 in Additional file 1). Rather than generating a binary classification, Align-GVGD categorizes missense substitutions into seven grades ordered from evolutionarily most likely (C0) to least likely (C65) [45]. Align-GVGD scored 14 of the rMSs as C0, with 12 patients versus 9 controls carrying a C0 rMS as their highest-grade *CHEK2 *variant. The OR for this grade of rMS was near 1.0 (OR, 1.39; 95% CI, 0.55 to 3.56) (Table 3). In contrast, five different rMSs scored as C65, with nine patients versus one control carrying a C65 rMS (again, as their highest-grade *CHEK2 *variant). The OR for C65 rMSs was 8.75 (*P *= 0.044) (Table 3). Exploiting the intrinsic ordering of the Align-GVGD grades, we performed a logistic regression test for loglinear OR trends across noncarriers and carriers of the seven grades of rMSs. This test yielded a lognormal OR increase of 0.33/grade (*P*_trend _= 0.0055) (Table 4). Thus the statistical evidence in favor of pathogenicity from the trend test was stronger than that generated by either the binary test over all the missense substitutions or the test for any individual grade of missense substitution. These results include adjustments for age category, study center and ethnic group. Neither the removal of the study center nor the inclusion of interactions between center and ethnic group changed the first two digits of these estimates. The interaction terms did not significantly improve the model fit (*P *= 0.18) and were omitted. While removing the study center did not significantly reduce the goodness of fit (*P *= 0.12), this adjustment was retained on the grounds of prior plausibility.

**Table 4:** Results from logistic regression tests for loglinear odds ratio trends^a^

	Loglinear OR regression coefficient (95% CI) and *P *value	
	
Grouping of rMS and/or T+SJV	Crude	**Adjusted**^**b**^
rMS only (that is, excluding T+SJV)	0.35 (0.12 to 0.58)	0.33 (0.09 to 0.55)
(note that C65 is grade 7)	*P *= 0.0029	*P *= 0.0055
		
C65 rMS and T+SJV	0.28 (0.14 to 0.43)	0.29 (0.14 to 0.43)
pooled in grade 7	*P *= 0.00013	*P *= 0.000088
		
C65 rMS in grade 7 and	0.26 (0.12 to 0.39)	0.26 (0.13 to 0.40)
T+SJV in grade 8	*P *= 0.00017	*P *= 0.00011

^a ^OR, odds ratio; 95% CI, 95% confidence interval; rMS, rare missense substitution; T+SJV, protein-truncating variants plus splice junction variant; ^b^Adjusted for race or ethnicity (Caucasian, East Asian, African American or Latina), study center and age as categorical variables.

We emphasize that our preplanned rMS analysis was based on rMS grading using Align-GVGD with a *CHEK2 *protein multiple sequence alignment having an average of at least three substitutions per position and in which the farthest diverged sequence was from the (deuterostomate) sea urchin (*Strongylocentrotus purpuratus*). Our analysis thus conformed to the conditions under which Align-GVGD was calibrated and was used to grade missense substitutions in *ATM *[7,45]. In addition to the pre-planned Align-GVGD analysis, we carried out corresponding analyses on the basis of rMS grading with SIFT [41,42] and PolyPhen-2 [46,47]. With SIFT, we set up three rMS grades: (1) the program's standard likely neutral grade of SIFT score >0.05, (2) a likely deleterious grade of 0.05 ≥ SIFT score ≥ 0.01, and (3) a more likely deleterious grade of SIFT score 0.00. Using a *CHEK2 *alignment in which the farthest diverged sequence was from the (protostomate) fruit fly (*Drosophila melanogaster*), which reached SIFT's median sequence conservation score threshold of 3.25, the OR for the SIFT score 0.00 grade was 3.03 and the logistic regression trend test gave *P*_trend _= 0.012 (Table 3). Using the slightly less informative alignment in which the most diverged sequence was from the sea urchin, the logistic regression trend test gave *P*_trend _= 0.014 (data not shown). PolyPhen-2 uses a combination of its own precompiled protein multiple sequence alignments and crystal structure information to score missense substitutions. Using PolyPhen-2, we also set up three rMS grades: (1) the program's standard "Benign" grade, (2) its standard "Possibly Damaging" grade, and (3) its standard "Probably Damaging" grade. The OR for the Probably Damaging grade was 3.87, and the logistic regression trend test gave *P*_trend _= 0.0070. The rMS grades obtained with SIFT and PolyPhen-2 are also included in Supplementary Table S1 in Additional file 1.

One question that arises from this approach to missense substitution analysis is whether the rMSs that drive the difference between patients and controls are truly evolutionarily unlikely, which is shorthand for "subject to purifying selection such that they are disproportionately unlikely ever to become fixed as major alleles." To address this question, we waited until after our primary protein multiple sequence alignment had been created and the rare human missense substitutions had been scored, then we assembled an additional mammalian *CHEK2 *gene model (from Guinea pig, *Cavia porcellus*). Insertion of the *C. porcellus *CHK2 sequence into our alignment and comparison with the other placental mammalian CHK2 sequences revealed 34 *C. porcellus*-specific amino acid substitutions (that is, apparently wild-type *C. porcellus *CHK2 amino acid residues that differ from the residues present at that position in the other placental mammalian CHK2 sequences). We then scored these residues with Align-GVGD as if they were amino acid substitutions in the human *CHEK2 *sequence. All 34 scored C0, the most evolutionarily likely grade and the grade that contributes least to the difference that we observe between breast cancer patients and controls. Simulating and scoring all possible single-nucleotide substitutions to the canonical human *CHEK2 *cDNA sequence, we found that 57.2% of possible missense substitutions are C0. Taking into account differing probabilities of these substitutions due to their underlying sequence contexts as estimated by dinucleotide substitution rate constants [54], 58.6% of a random draw of missense substitutions would be C0. Therefore, ignoring the effects of purifying selection, the probability that 34 of 34 *C. porcellus*-specific substitutions would be C0 is ~0.586^34 ^= 1.3 × 10^-8^. Thus selection acts against the rMSs of grade >C0. As these grades have sequentially increasing leverage (toward C65) on the test for trends, evolutionarily unlikely rMSs indeed drive the observed difference between patients and controls.

### Combined evidence

Looking forward to candidate gene studies, it could be useful to combine evidence from both T+SJVs and rMSs. The loglinear OR trend test provides a simple mechanism by which to achieve this end: observations of T+SJVs can either be combined with observations of the highest grade of missense substitutions (C65s) or we can add an eighth (even higher) carrier grade for the T+SJVs. For this data set, combining T+SJVs and C65 rMSs in grade 7 appeared to be slightly more effective: lognormal OR increased by 0.29/grade (*P*_trend _= 8.8 × 10^-5^) as opposed to 0.26/grade (*P*_trend _= 1.1 × 10^-4^) with the alternative approach. The important point is that the data were less compatible with chance when combined than when they were considered as either T+SJVs or rMSs alone.

### Extrapolation to pathway and whole-exome case-control mutation-screening projects

Massively parallel sequencing has evolved to the point where it is being used to identify susceptibility genes for rare diseases, and one can imagine study designs where it could be used to identify or characterize intermediate-risk susceptibility genes for common diseases. Using rare variant carrier frequencies of 0.0045, 0.0018, 0.00021*, 0.00011*, 0.00090 and 0.0027 for the rMS grades C15, C25, C35*, C55*, C65 and T+SJV, respectively, as well as ORs of 1.82, 2.47, 3.74*, 7.24*, 8.75 and 6.18 for the same series of grades, we estimated the number of participants required for a reasonably powered many-gene case-control mutation-screening study. (Note that these frequency and OR values were taken or calculated directly from Tables 3 and 4 unless marked with an asterisk; marked values were estimated from the lognormal OR regression coefficient given in Table 4 and the number of observations in patients.) Setting a Bonferroni-adjusted *P *value threshold of 0.0005 for a study of the ~100 genes in the DNA double-stranded break repair and allied cell cycle checkpoint pathways, we estimate that ~2,000 cases and a similar number of controls would be required for 80% power in a combined analysis of T+SJVs and rMSs (Table 5). An analysis based on T+SJVs alone would require 3,400 each of patients and controls, and an analysis based on rMSs alone would require 4,700 each of patients and controls. Setting a *P *value threshold of 2.5 × 10^-6^, which might be considered appropriate for a whole-exome study, 3,350 each of patients and controls would be required for 80% power.

**Table 5:** Number of patients and frequency-matched controls required for various scales of future intermediate-risk gene case-control mutation-screening studies^a^

Study scale	Single genes	**Whole pathways**^**b**^	**Whole exome**^**c**^
Type I error	0.05	0.0005	2.5 × 10^-6^
Power	0.80	0.80	0.80
rMS alone, *n*	1,975	4,700	7,725
T+SJV alone, *n*	1,425	3,400	5,600
rMS plus T+SJV, *n*	850	2,025	3,350

^a ^rMS, rare missense substitution; T+SJV, protein-truncating variants plus splice junction variant; ^b ^Calculated for 100 genes, approximately the gene count of DNA double-stranded break repair and associated cell cycle checkpoints; ^c^Calculated for 20,000 genes.

## Discussion

That protein-truncating variants in *CHEK2 *confer a moderately increased risk of breast cancer is well established. The OR that we observed for T+SJVs is numerically somewhat higher than that reported in the 2004 CHEK2 Breast Cancer Case-Control Consortium study of *c.1100delC *[50], but not significantly, as our 95% CIs do include the point estimate from that study. Moreover, as previous studies have observed higher ORs for *c.1100delC *in familial versus sporadic cases and in early-onset versus later-onset cases [9,50], we should expect that this study's focus on early-onset breast cancer cases with oversampling of familial cases would result in relatively high OR estimates.

Previous studies have shown that some *CHEK2 *missense substitutions are pathogenic, but the scale of their contribution to breast cancer susceptibility relative to that of T+SJVs is not known. Although we hesitate to extrapolate our current data to true population-attributable risks (within the age groups that we sampled) or familial relative risks, the data do provide a basis on which to compare the relative contributions of these two classes of variants. Working from the control carrier frequencies and the OR point estimates (adjusted for race or ethnicity, study center, and age) observed from the population-based Breast CFR sample series, we calculate attributable fractions of 0.014 for T+SJVs as compared with 0.015 for the sum of C15-C65 rMSs. In addition, we calculate a familial relative risk among first-degree relatives of 1.036 for T+SJVs as compared with 1.033 for a product across the C15-C65 rMSs. Thus, as a first approximation, the attributable fractions and familial relative risks of truncating variants and rare missense substitutions are virtually identical. It is important to remember that these attributable fraction and familial relative risk point estimates are inflated compared with those that would be obtained from a population-based study that included patients diagnosed in their 70s or older. In addition, as more than 25% of the T+SJVs observed in this study were nonsense and frame shift mutations other than *c.1100delC*, these data also speak to the importance of full open reading frame mutation screening to observe the majority of genetically relevant sequence variants in this cancer susceptibility gene.

Several of the missense substitutions observed in this study have been subjected to functional assays in one or more published works. For the 14 missense substitutions that Align-GVGD scored C0 and which we would consequently predict to be neutral or nearly so, assay results have been reported for 4 (p.P85L, p.R137Q, p.R180H and p.T323P). Using a *Saccharomyces cerevisiae *Rad53 complementation assay, Shaag *et al. *[22] found that *p.P85L *is equivalent to wild-type *CHEK2*. While Bell *et al. *[55] found this allele to have modestly reduced activity in an *in vitro *kinase function assay, both Bell *et al. *and Shaag *et al. *concluded that the allele is effectively neutral. Sodha *et al. *[39] assayed the *p.R137Q *allele and found that it encodes a protein with normal stability and normal response to DNA damage. Bell *et al. *[55] also assayed the *p.R137Q *allele and found that it has normal kinase activity. In addition, Sodha *et al. *[39] assayed the *p.R180H *allele and found that it encodes a protein with slightly reduced stability but normal response to DNA damage. Thus existing functional assay results for these three variants are consistent with their being either neutral or at most weakly pathogenic. Wu *et al. *[56] found the fourth C0 substitution, p.T323P, to have moderately reduced autophosphorylation and Cdc25C kinase activity. Classification of this substation as C0 is probably a true Align-GVGD error, because the crystal structure of the protein reveals that T323 is located in an α-helix, which would not typically be permissive of substitution to proline. The algorithmic problem is that the atomic composition and polarity of proline (the amino acid side chain characteristics considered by the original Grantham difference [57] and Align-GVGD are atomic composition, polarity and volume) are intermediate between those of threonine and isoleucine, which are the two amino acids observed at position 323 in our alignment. The consequence is that proline is only slightly outside the range of variation represented by these two wild-type residues and is consequently predicted to be neutral or nearly so. Although unpublished, misclassification of substitutions to proline that map within an α-helix is a problem that we have observed before and is an obvious issue to bear in mind when considering missense substitution analyses made using Align-GVGD. p.I157T is perhaps the most interesting of the substitutions observed in our study that have been subjected to functional assays. Align-GVGD scores the variant as C15, indicative of modest evidence in favor of pathogenicity. Initially, Lee *et al. *[58] found that kinase activity of the *p.I157T *allele was comparable to the wild type. More recent studies have reported that the allele is at least partially defective in dimerization and autophosphorylation, binding and phosphorylating Cdc25, and binding *BRCA1 *[59-62]. In populations in which *p.I157T *and *c.1100delC *are both present at appreciable frequencies and have been subject to independent risk estimates, *p.I157T *does appear to confer increased risk of breast cancer, but the OR or penetrance associated with the missense substitution appears to be more modest than that associated with the frame shift *c.1100delC *[63]. At the other end of the spectrum, of the five C65 substitutions that we observed, only one, p.R117G, has been subjected to functional assays. Summing across several studies, the protein encoded by this allele is phosphorylated by ATM in response to DNA damage, shows slightly to markedly reduced autophosphorylation, probably fails to oligomerize and has severely compromised kinase activity toward Cdc25C [39,56,62]. Therefore, the *p.R117G *allele encodes a functionally defective protein and is in all likelihood pathogenic. Thus, for the missense substitutions that were observed in our mutation-screening study and subjected to functional assays, there is a qualitative trend toward agreement between the Align-GVGD classification and the functional assay result, consistent with the trend in ORs that we observed across the Align-GVGD-defined ordered series of missense substitution grades. However, since concordant results between *in silico *assessments and functional assays are not yet considered sufficient for formal clinical classification of missense substitutions observed in *BRCA1 *and *BRCA2 *[64-66], it does not appear that the state-of-the-art of *CHK2 *functional assays has reached the point at which concordant results from an *in silico *assessment and a functional assay would be sufficient for clinically relevant classification of a *CHEK2 *missense substitution.

The genetic results described in this work, combined with the above functional assay summary, have implications for potential clinical genetic susceptibility tests that might include *CHEK2 *and other genes with similar mutation profiles. In the 2003 American Society of Clinical Oncology Policy Statement Update on Genetic Testing for Cancer Susceptibility, the second and third "indications for genetic testing for cancer susceptibility" were that "2) the genetic test can be adequately interpreted, and 3) the test results will aid in diagnosis or influence the medical or surgical management of the patient or family members at hereditary risk of cancer" (pp. 2398) [67]. With regard to the third criterion, some investigators have argued that in the context of a high-risk family, the difference in risk between carriers and noncarriers of clearly pathogenic *CHEK2 *sequence variants is sufficient to justify a difference in cancer surveillance strategies [68-70]. However, our results in addition to similar work regarding *ATM *[7,71] point toward an issue under the second criterion. If roughly one-half of the genetically relevant risk that the test can pick up actually resides in rare missense substitutions that will be considered unclassified variants at their initial detection, it may not currently be possible to adequately interpret the test results. Therefore, while it is now technically feasible to design a massively parallel sequencing-based test that can accurately and relatively inexpensively identify mutations in a panel of breast cancer susceptibility genes that includes *ATM *and *CHEK2 *[72], it may be inappropriate to introduce such a test into widespread use before a clinically validated method of assessing unclassified missense substitutions in these genes has been developed.

The rare missense substitution analysis model combining Align-GVGD with the logistic regression test for trends grew out of the *in silico *analysis of missense substitutions that has now become a standard component in the integrated evaluation of unclassified variants in *BRCA1 *and *BRCA2 *[65,73]. We proposed the model on the basis of clinical *BRCA1 *and *BRCA2 *mutation-screening data and then demonstrated its effectiveness by an analysis of *ATM *case-control mutation-screening data [7,45]. Thus the *CHEK2 *analysis presented here stands as a methodological confirmation of our approach to the inclusion of rare missense substitution data in case-control mutation-screening studies. The logistic regression test for trends that we used also provides a simple approach to combining evidence from rare missense substitutions with evidence from protein-truncating sequence variants to build a more complete and statistically powerful approach to assessing case-control mutation-screening data than would be afforded by either method alone. From a technological perspective, we can envision combining exon capture and massively parallel sequencing to extend case-control mutation screening to entire biochemical pathways and beyond. On the basis of our *post hoc *power calculations, at least 2,000 patients and 2,000 controls would be required for a whole pathway (such as DNA double-stranded break repair and allied cell cycle checkpoints) study, and 3,300 patients and 3,300 controls would be required to undertake a whole-exome study. On the one hand, these numbers could be an underestimate because *CHEK2 *might be among the most important (in terms of familial relative risk) of the intermediate-risk class of breast cancer susceptibility genes. On the other hand, it could turn out that a test based on observations of evolutionarily unlikely sequence variants has an intrinsically lower false-positive rate than anonymous marker GWASs and consequently would not require a full Bonferroni multiple testing correction to reasonably constrain the rate of false-positive results.

## Conclusions

This case-control mutation-screening study of *CHEK2 *shows that the gene harbors many different rare pathogenic sequence variants, a substantial proportion of which are missense substitutions. From a clinical perspective, the risk of breast cancer conferred by some pathogenic sequence variants in *CHEK2 *may be great enough to be of use in a clinical cancer genetics setting, and we note that the technical capability of offering a multigene breast cancer susceptibility testing panel at relatively low per gene laboratory cost is in place. Yet, our results with both *CHEK2 *and *ATM *suggest that such a test would create a severe burden of unclassified missense substitutions and that a large fraction of the genetically relevant risk would reside in those unclassified missense substitutions. Paradoxically, on the basis of the research perspective of susceptibility gene identification and characterization, this study validates our approach to the analysis of rare missense substitutions observed during case-control mutation screening and provides a method to combine data from protein-truncating variants and rare missense substitutions into a one degree of freedom per gene test.

## Abbreviations

Breast CFR: Breast Cancer Family Registry; GWAS: genome-wide association study; IRB: institutional review board; OR: odds ratio; PCR: polymerase chain reaction; rMS: rare missense substitution; SNP: single-nucleotide polymorphism; T+SJV: truncating and splice junction variant; WGA: whole genome amplification.

## Competing interests

The authors declare that they have no competing interests.

## Authors' contributions

FLCK contributed to study design, led the laboratory team and helped to draft the manuscript. FL contributed to study design, led the data analysis and helped to draft the manuscript. FD contributed to the mutation screening and data analysis and helped to refine the laboratory platform. MV contributed to the sequence alignment and data analysis. CV was responsible for data management throughput for the project and helped to refine the laboratory platform. DB contributed to the sequence alignment and method for analysis of rare missense substitutions. GD contributed to the mutation screening and data analysis and helped to refine the laboratory platform. NF contributed to the mutation screening and data analysis and helped to refine the laboratory platform. SMC contributed to the mutation screening and data analysis and helped to refine the laboratory platform. NR contributed to the mutation screening and data analysis and helped to refine the laboratory platform. TND contributed to the sequence alignment and data analysis. AT contributed to statistical analyses and helped to draft the manuscript. GBB contributed to statistical analyses and helped to draft the manuscript. JLH was responsible for patients gathered through the University of Melbourne and helped to draft the manuscript. MCS contributed to study design and contributed to the management of samples obtained through the University of Melbourne. ILA was responsible for patients gathered through Cancer Care Ontario. EMJ was responsible for patients gathered through the Northern California Cancer Center (now the Cancer Prevention Institute of California). SVT was responsible for overall study design, contributed to data analysis and helped to draft the manuscript. All authors read and approved the final manuscript.

## Supplementary Material

Additional file 1 **Supplementary Tables S1 and S2**. Supplementary Table S1: Missense, nonsense, frame shift, and splice junction variants. Supplementary Table S2: CHEK2 protein multiple sequence alignment characterization. Click here for file
