# Supplementary material for: Rare, evolutionarily unlikely missense substitutions in CHEK2 contribute to breast cancer susceptibility: results from a breast cancer family registry case-control mutation-screening study
Source: Breast Cancer Res. 2011 Jan 18;13(1):R6. doi: 10.1186/bcr2810 (PMC3109572; doi:10.1186/bcr2810)
Supplement: Additional file 1 — Supplementary Tables S1 and S2. Supplementary Table S1: Missense, nonsense, frame shift, and splice junction variants. Supplementary Table S2: CHEK2 protein multiple sequence alignment characterization. [file bcr2810-S1.PDF]

Supplementary table 1. Missense, nonsense, frameshift, and splice junction variants

			dbSNP rs number (if any)	Align-GVGD Spur[1]	SIFT Dmel[2]	PolyPhen2 PolyPhen-HumDiv	Number of carriers control	case
Missense substitutions								
c.14C>T	p.S5L			C0	0.06	benign	1	2
c.74T>C	p.V25A			C0	0.13	benign	0	1
c.254C>T	p.P85L	†	rs17883862	C0	0.00	Poss. Damaging	3	1
c.349A>G	p.R117G	†	rs28909982	C65	0.00	Prob. Damaging	1	3
c.410G>A	p.R137Q	¥		C0	0.38	Poss. Damaging	0	1
c.470T>C	p.I157T	†	rs17879961	C15	0.07	benign	1	2
c.538C>T	p.R180C	†	rs77130927	C25	0.01	Poss. Damaging	0	3
c.539G>A	p.R180H	¥		C0	0.06	Prob. Damaging	1	0
c.575C>T	p.S192L			C15	0.01	Prob. Damaging	0	1
c.663C>G	p.I221M			C0	0.11	Poss. Damaging	0	1
c.688G>T	p.A230S			C15	0.03	benign	1	0
c.715G>A	p.E239K	†	rs121908702	C15	0.02	Poss. Damaging	0	2 *
c.727T>C	p.C243R			C0	0.01	Prob. Damaging	1	0
c.751A>T	p.I251F	¥		C15	0.01	Prob. Damaging	1	0
c.911T>C	p.M304T			C25	0.10	Prob. Damaging	0	1
c.917G>C	p.G306A			C0	0.06	Prob. Damaging	0	1
c.931G>A	p.D311N			C0	0.41	benign	0	1
c.967A>C	p.T323P	¥		C0	0.12	Prob. Damaging	0	1
c.1036C>T	p.R346C			C65	0.01	Prob. Damaging	0	3
c.1037G>A	p.R346H			C25	0.00	Prob. Damaging	0	1 *
c.1054A>T	p.N352Y			C65	0.00	Prob. Damaging	0	1
c.1111C>T	p.H371Y			C0	0.19	benign	1	2
c.1182A>T	p.E394D			C35	0.00	Prob. Damaging	0	1 *
c.1216C>T	p.R406C			C15	0.00	Poss. Damaging	0	1
c.1253T>G	p.F418C			C55	0.00	Prob. Damaging	0	1
c.1276C>T	p.P426S			C65	0.00	Prob. Damaging	0	1
c.1312G>T	p.D438Y	¥		C25	0.01	Poss. Damaging	2	2
c.1313A>G	p.D438G			C15	0.04	Prob. Damaging	0	1
c.1336A>G	p.N446D			C0	0.60	benign	1	0
c.1343T>G	p.I448S	†	rs17886163	C15	0.69	benign	2	8 *
c.1427C>T	p.T476M			C15	0.00	Prob. Damaging	0	1
c.1451C>T	p.P484L			C65	0.01	Prob. Damaging	0	1
c.1534C>G	p.L512V	†	rs17882942	C0	0.95	benign	0	1
c.1556G>T	p.R519L			C0	0.07	Poss. Damaging	1	0
Nonsense, truncating, and splice junction variants								
c.283C>T	p.R95X			na	na		0	1
c.405delA	p.K135fs			na	na		0	1
c.823G>T	p.E275X			na	na		0	1
c.1100delC	p.T367fs	¥		na	na		3	11
c.1138delCT	p.L380fs			na	na		0	1
c.1263delT	p.L421fs			na	na		0	1
c.1528C>T	p.Q510X			na	na		0	1

na Not applicable.
1 Using the *CHEK2* sequence alignment through *S. purpuratus* (sea urchin).
2 Using the *CHEK2* sequence alighnment through *D. melanogaste*r (fruitfly).
† Present in dbSNP as of 9 November, 2010.
¥ Not present in dbSNP, but reported in one or more manuscripts identified with the PubMed query <CHEK2 AND missense> on 9 November, 2010.
* Missense substitutions observed in an individual who carried another rare variant of interest. Specifically, one case carried p.I448S (C15, S=0.69, PolyPhen "Benign") plus p.E394D (C35, S=0.00, PolyPhen "Probably Damaging"), and one case carried p.E239K (C15, S=0.02, PolyPhen "Possibly Damaging") plus p.R346H (C25, S=0.00, PolyPhen "Probably Damaging). In the logistic regressions, these two subjects were placed in the category corresponding to their higher Align-GVGD grade (or lower SIFT score, or more damaging PolyPhen designation) missense substitution.

Supplementary Table 2. CHEK2 protein multiple sequence alignment characterization

Sequence source	Sequence length	Ave. number of substitutions per position	SIFT: median sequence conservation score	Percent amino acid sequence identity in pairwise comparison Hsap	Mmus	Cfam	Btau	Lafr	Mdom	Oana	Ggal	Xlae	Drer	Spur	Cint	Dmel
Hsap	543	na	na	1.00												
Mmus	546	nm	nm	0.83	1.00											
Cfam	544	nm	nm	0.89	0.83	1.00										
Btau	534	nm	nm	0.87	0.82	0.92	1.00									
Lafr	565	nm	nm	0.86	0.81	0.88	0.87	1.00								
Mdom	531	nm	nm	0.76	0.73	0.76	0.77	0.76	1.00							
Oana	537	nm	nm	0.74	0.71	0.74	0.73	0.72	0.76	1.00						
Ggal	522	nm	nm	0.69	0.66	0.68	0.69	0.67	0.71	0.71	1.00					
Xlae	517	nm	nm	0.61	0.60	0.61	0.62	0.60	0.65	0.65	0.65	1.00				
Drer	503	2.46	3.71	0.52	0.51	0.51	0.52	0.50	0.55	0.53	0.56	0.53	1.00			
Spur	496	3.08	3.54	0.48	0.49	0.47	0.48	0.46	0.48	0.48	0.49	0.47	0.44	1.00		
Cint	557	3.97	3.33	0.39	0.39	0.39	0.40	0.39	0.39	0.40	0.38	0.38	0.38	0.41	1.00	
Dmel	459	4.86	3.20	0.35	0.36	0.36	0.36	0.34	0.37	0.36	0.35	0.37	0.36	0.38	0.33	1.00

na Not applicable.

nm Not measured. These measurements were conducted by sequentially removing sequences from the "complete" alignment and then performing the analysis indicated.   Thus there was no need to continue after the alignment failed to meet either informativeness criterion.

Species abbreviations: Hsap, Homo sapiens (human); Mmus, Mus musculus (mouse); Cfam, Canis familiaris (dog); Btau, Bos taurus (cow); Lafr, Loxodonta africana (elephant); Mdom, Monodelphis domestica (opossum); Oana, Ornithorhynchus anatinus (platypus); Ggal, Gallus gallus (chicken); Xlae, Xenopus laevis (frog); Drer, Danio rerio (zebrafish); Spur, Strongylocentrotus purpuratus (purple sea urchin); Cint, Ciona intestinalis (tunicate); Dmel, Drosophila melanogaster (fruitfly).
